# Ilio-Rectal Fistula

**Published:** 1900-04

**Authors:** S. H. Swain

**Affiliations:** Decatur, Ill.


### REPORTS OF CASES.

### ILIO-RECTAL FISTULA.

By S. H. Swain, V.S.,
DECATUR, ILL.

The subject of this article was a splendid specimen of the equine family, a bay colt, aged about six months, sex male, owned by James Dickey, of Voorhies, Ill.

The history of this case showed that while the mother was being driven on the road, in shafts, the colt suddenly stopped in front of the mare, and by so doing had received an injury from the point of the shaft in the region of the anus, and that considerable tumefaction followed in the right sacral region, with a discharge of pus from the rectum and later by atrophy of the sacral muscles. A local doctor was consulted, who attempted treatment by injections per rectum and blisters over the tumefaction in the sacral region. After an unsuccessful attempt, lasting for a period of about two months, the colt was sent to me for treatment, and on making a rectal exploration a fistula was discovered extending in the direction of the inferior sacro-iliac region, with communication in the rectum, about four inches anterior to the anal opening.

On this discovery an operation was decided upon ; an incision was then made in the depression between the anus and point of ischium on the right side of the anus and exterior to the sphincter ani. Then the left hand was introduced into the rectum and a probe passed into the fistulous opening sinus ; then a blunt instrument in the right hand was introduced into the incision above mentioned, and pushed forward parallel with the rectum until an intersection was made with the fistulous sinus containing the probe ; an external drain was thus successfully made without serious injury to the patient.

Treatment was now begun, and consisted of daily injections into the fistulous tract of hydrogen peroxide until one pound had been used, then the peroxide was discontinued, and the following preparation was substituted : Zinc chloride, 2 drachms ; tr. myrrh, 4 ounces ; distilled water, 60 ounces. This was mixed and injected once or twice daily by means of a small syringe with a long slender point. This treatment was continued from day to day, with a gradual slow improvement, it becoming necessary to frequently dilate the external drain for several inches in depth in order to secure healing from the bottom, with the result that after a continuous, patient treatment of this case for a period of nearly three months the patient was discharged from our hospital completely cured and without a blemish.

CASE II. was a fine grade Percheron mare, about ten years old, and weighed about thirteen hundred pounds, owned by George Short, of Emery, Ill., and came under my observation some twenty years ago.

The history, as given by the owner, showed that the mare had difficulty in defecation for quite a while prior to my visit, and also had shown colicky symptoms and had been raked per rectum by the owner without discovering the true nature of the trouble, and with only temporary relief ; then the owner decided to call me in consultation.

On my arrival and after learning the history of the case a rectal examination was made, and the entire pelvic outlet was found to be completely blocked with feces, a removal of which revealed an enormous pocket occupying the entire ilio-rectal space and containing, I think, not less than one peck of closely compressed fecal matter. This was removed and the pocket carefully examined for our own information, and then an unfavorable prognosis was pronounced, and the owner was advised to destroy the mare, which was done at the next occurrence of the impaction.

It is the opinion of the writer that this case was at one time very similar to the one described in the colt, and could without doubt have been treated with complete success if taken in time. On account of the unusual occurrence of this accident and a lack of literature on the subject, I have thought a report of these two cases would be of interest to the profession.

